# A Review of Indirect Tool Condition Monitoring Systems and Decision-Making Methods in Turning: Critical Analysis and Trends

**DOI:** 10.3390/s21010108

**Published:** 2020-12-26

**Authors:** Mustafa Kuntoğlu, Abdullah Aslan, Danil Yurievich Pimenov, Üsame Ali Usca, Emin Salur, Munish Kumar Gupta, Tadeusz Mikolajczyk, Khaled Giasin, Wojciech Kapłonek, Shubham Sharma

**Affiliations:** 1 Mechanical Engineering Department, Technology Faculty, Selcuk University, Selçuklu, 42130 Konya, Turkey; mkuntoglu@selcuk.edu.tr; 2 Mechanical Engineering Department, Engineering and Architecture Faculty, Selcuk University, Akşehir, 42130 Konya, Turkey; aaslan@selcuk.edu.tr; 3 Department of Automated Mechanical Engineering, South Ural State University, Lenin Prosp. 76, 454080 Chelyabinsk, Russia; munishguptanit@gmail.com; 4 Mechanical Engineering Department, Engineering and Architecture Faculty, Bingöl University, 12000 Bingöl, Turkey; ausca@bingol.edu.tr; 5 Department of Metallurgical and Materials Engineering, Selcuk University, Selçuklu, 42130 Konya, Turkey; esalur@selcuk.edu.tr; 6 Key Laboratory of High Efficiency and Clean Mechanical Manufacture, Ministry of Education, School of Mechanical Engineering, Shandong University, Jinan 250100, China; 7 Department of Production Engineering, UTP University of Science and Technology, Al. Prof. S. Kaliskiego 7, 85-796 Bydgoszcz, Poland; tami@utp.edu.pl; 8 School of Mechanical and Design Engineering, University of Portsmouth, Portsmouth PO1 3DJ, UK; Khaled.Giasin@port.ac.uk; 9 Department of Production Engineering, Faculty of Mechanical Engineering, Koszalin University of Technology, Racławicka 15-17, 75-620 Koszalin, Poland; wojciech.kaplonek@tu.koszalin.pl; 10 Department of Mechanical Engineering, IKG Punjab Technical University, Jalandhar-Kapurthala Road, Kapurthala, Punjab 144603, India; shubham543sharma@gmail.com

**Keywords:** indirect tool condition monitoring systems, turning, machining, vibration, cutting force, acoustic emission, temperature, current, industry 4.0

## Abstract

The complex structure of turning aggravates obtaining the desired results in terms of tool wear and surface roughness. The existence of high temperature and pressure make difficult to reach and observe the cutting area. In-direct tool condition, monitoring systems provide tracking the condition of cutting tool via several released or converted energy types, namely, heat, acoustic emission, vibration, cutting forces and motor current. Tool wear inevitably progresses during metal cutting and has a relationship with these energy types. Indirect tool condition monitoring systems use sensors situated around the cutting area to state the wear condition of the cutting tool without intervention to cutting zone. In this study, sensors mostly used in indirect tool condition monitoring systems and their correlations between tool wear are reviewed to summarize the literature survey in this field for the last two decades. The reviews about tool condition monitoring systems in turning are very limited, and relationship between measured variables such as tool wear and vibration require a detailed analysis. In this work, the main aim is to discuss the effect of sensorial data on tool wear by considering previous published papers. As a computer aided electronic and mechanical support system, tool condition monitoring paves the way for machining industry and the future and development of Industry 4.0.

## 1. Introduction

As soon as the machining process starts, the contact between tool and workpiece leads to various events for chip removing and generating a fresh surface [1,2]. The relative movement between tool and workpiece causes deterioration and is commonly known as tool wear (TW) [3]. Several types of TW become active during cutting operation; however, flank wear (FW) is accepted as the main tool life criteria and will be the research subject of this paper [4].

Prediction of tool life is a serious issue because of the non-linear and dynamic structure of cutting operations [5]. The complex relationship between parameters is revealed to consider each operation in itself as a result of dynamic interaction which occurs instantly. Sensors act as information sources situated around the cutting area and transmit the obtained data as rapid as possible [6]. Since sensor signals show different behavior according to machining conditions and TW, then it becomes hard to state the tool condition. For this reason, multiple sensor systems are preferred for confirmation of the different sensor signals.

Tool condition monitoring system (TCMS) provides online monitoring of cutting operation bringing several advantages such as prediction of tool life, preventing catastrophic failures and increasing the productivity and component quality [7]. Cutting tool has a specialized form produced with a certain material to work under harsh conditions [8]. Basically, monitoring of cutting tool condition avoids unexpected changes, reduces downtime, obtains desired workpiece dimensions and eventually reduces production costs and labor [9]. In this manner, FW becomes significant as the first tool life criteria and optimization of FW is highly necessary depending on optimal cutting conditions [3].

With the increasing prospects from manufacturers such as precise and punctual delivery which is basically associated with determining the optimum machining environment, several TCM approaches supported by different methods such as deep learning have been proposed [10]. In order to overcome the challenges in the future, a new perspective is required to integrate the TCMS into Industry 4.0 which is defined as the transformation from machine based manufacturing into digital manufacturing [11,12]. The idea covers non-human manufacturing environment making enabling it with internet of things and communication of machines [13]. It is in common with the TCMS and Industry 4.0 target. As known, for better evaluation and monitoring of tool condition, numerous factors should be considered in each operation. Therefore, requirements exists such a which system can correlate the turning variables and tool wear, capable of sensing the changes bad or good and their effects on tool wear more or less. That is why indirect TCMS and decision-making methods in turning are discussed trying to demonstrate the trends.

The main aim of this study is to summarize the indirect TCMS in detail. The subdivisions of these systems, past studies about TCMS in turning and the relationship of different sensor signals with FW will be investigated. These systems play a fundamental role for Industry 4.0 and heads for cost optimized manufacturing. An increasing trend named hybrid approaches motivates researchers to combine different methods for robust design and accurate evaluation of results [14,15,16]. TCMS allows for the research complex relations between turning parameters and process variables if it is accommodated to these systems traditional methods, i.e., analysis of variance, Taguchi, artificial neural network and fuzzy logic or a newly developed method, i.e., the bees’ algorithm [17]. This approach enables to correlate sensorial data with TW and to understand the progression of wear without stopping the operation.

In this study, TCMS and its subsection, indirect TCMS, are summarized, supported with the published literature covering their structure and main approaches first. Then, sensors used in indirect TCMS such as cutting forces, AE, vibration, temperature, current, sound and image processing along with FW and SR are outlined with their advantages and disadvantages. Data acquisition and signal processing methods are evaluated considering decision-making approaches such as artificial neural networks, fuzzy logic, hidden Markov model, support vector machine and ANFIS. Lastly, critical analysis and trends are outlined to demonstrate the current developments in this field in the perspective of the authors.

## 2. Tool Condition Monitoring Systems

During more than 40 years of the studies about TCMS, the main focus has remained the same in terms of researchers and manufacturers: to recognize the tool state with several detection systems based on sensors and signal features. Since a single sensor had no capability to detect all or majority of the changes in an operation, multiple sensors needed to be applied in the past. The other side of this issue covers the software part for better understanding of the machining. In the first efforts in this field, Dornfeld and Pan [18] integrate AE for chip formation and Balakrishnan et al. [19] utilized both cutting force and AE together. After these studies, neural networks and pattern recognition based approaches were tried for decision making for real time monitoring in several papers [20,21,22]. From this point, commercial types of TCMS endeavored to integrate covering plenty of industrial companies [23]. Advanced TCMS have been extended with expedition of communication systems and the increase of internet from 2000 due to the possibility of purchasing sensor systems worldwide from the researchers in this field [24]. To date, comprehensive sensor technologies, signal processing and decision-making strategies have been applied, which will be further explained. Therefore, there was a striving in the long past of the TCMS to improve the machining operations integrating intelligent systems and to reach unmanned manufacturing with minimized error and high reliability.

Cost optimization and productivity became the main purpose in industry [25,26]. Breakdowns depend on tool breakage cause cutting tool-related downtime and expense which possess a significant place in overall cost [27]. In unmanned manufacturing, online monitoring of TW is necessary for productive usage of tool in lifetime and to prevent tool breakage. FW develops on the main cutting edge of a cutting tool as a result of the friction between cutting tool and workpiece [8]. Additional to determining desired tool life, FW affects directly SR and dimensional accuracy of workpiece. In the absence of TCMS, power consumption and manufacturing cost increase, dimensional accuracy, authority on process and sustainability decrease [28].

TCMS are examined in two different categories according to the approach of evaluation of TW in machine tools [29]. Direct methods require measuring the TW on cutting tool as area, length or width via an imaging technology. Main disadvantage of this method is to cause machine downtime and prolong manufacturing times [30]. Indirect methods on the other hand enable online monitoring of TW without intervention to process using sensor systems. Indirect methods are based on the measurement approach establishing a correlation between TW and sensor signals (CF, acoustic emission (AE), vibration, temperature, motor current/power, sound, SR). Some studies have been performed in the past which reviewed the TCMS from different viewpoints. The success of sensorial data in detecting TW is an aim in this study, primarily. In this context, TW types except for FW were not considered deliberately, and mostly preferred sensors and signal processing methods in TCMS in turning were investigated to outline the literature.

## 3. Indirect Tool Condition Monitoring Systems

**Figure 1:**
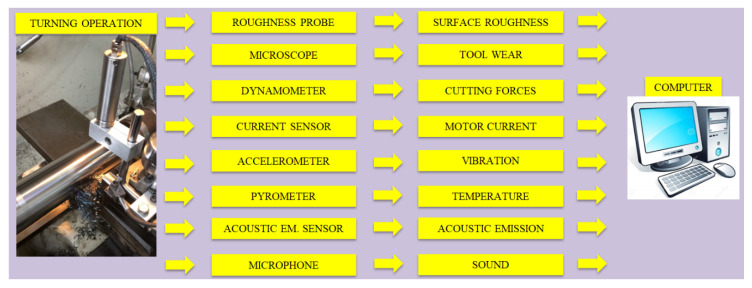
General outline of indirect tool condition monitoring system (TCMS) for turning.

**Figure 2:**
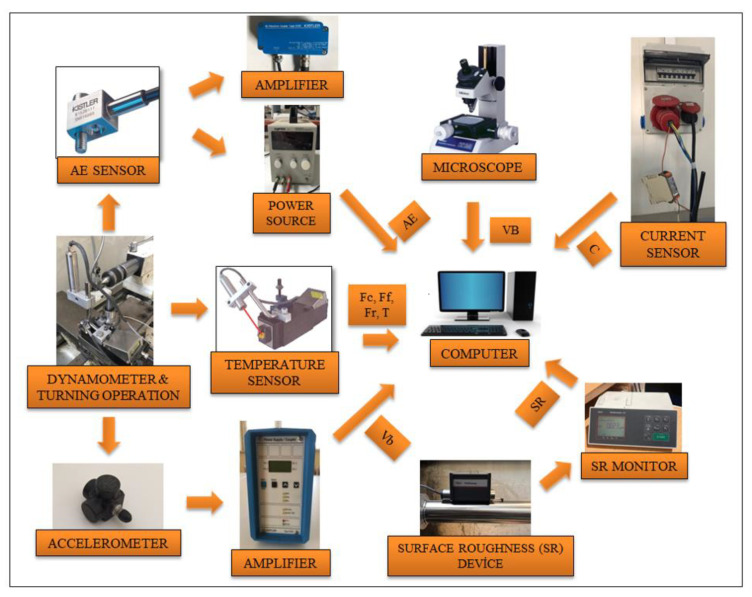
An example for an indirect tool condition monitoring system [5].

**Figure 3:**
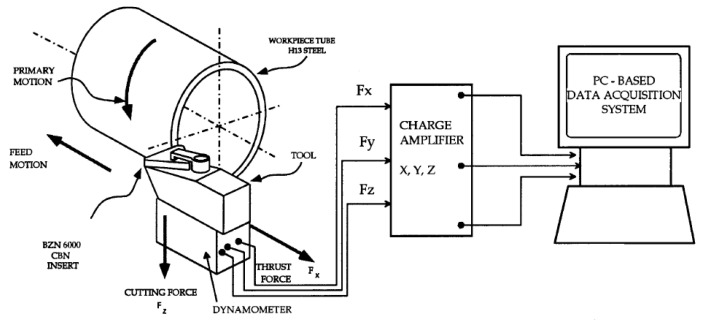
An example for an indirect tool condition monitoring system [35].

Monitoring of tool condition is a constraining and unstable process in machining operations [31]. TW is both inevitable and necessary for chip removing and manufacturing new product. With generating new surface, TW proceeds with time, and sudden changes occur which affect the wear progression more or less. Depending on chip formation, vibration occurrence, or inhomogeneity of material structure, some unintended contacts between tool and workpiece take place [32]. These unpredictable challenges make difficult to determine tool life precisely. The need for TCMS arises from the natural development of TW and mentioned difficulties. It was stated from [33] that it can be possible to arrange the tool position for determined contact conditions between tool and workpiece with prediction of TW. At this point, indirect TCMS presents a non-destructive, easy and sustainable approach without interference to machining system [34]. The main focus is to demonstrate and investigate the papers which have motive to associate the sensor signals with FW via indirect TCMS. As it is demonstrated in Figure 1, to evaluate TW with various measurement devices, it is preferred roughness probe to measure SR, dynamometer to measure cutting forces, current sensor to measure motor current, accelerometer to measure vibration, pyrometer to measure temperature, AE sensor to measure AE, microphone to measure sound. Moreover, Figure 2 and Figure 3 show examples of TCMS developed before to monitor FW in turning. In the aspect of indirect TCMS, two types of measurement become prominent to further evaluation of gathered data. The first one is online monitoring of various data performed with sensors such as vibration, AE, CF, temperature, sound and current. The data collection from these sensors continues throughout operation without stoppage. The other type of measurement is off-line monitoring namely SR and TW which needs to be stopped of machining. In Table 1 and Table 2, publications used different methods in indirect tool condition monitoring system, and publications that used decision making methods in indirect tool condition monitoring system are listed respectively.

### 3.1. Cutting Forces

**Figure 4:**
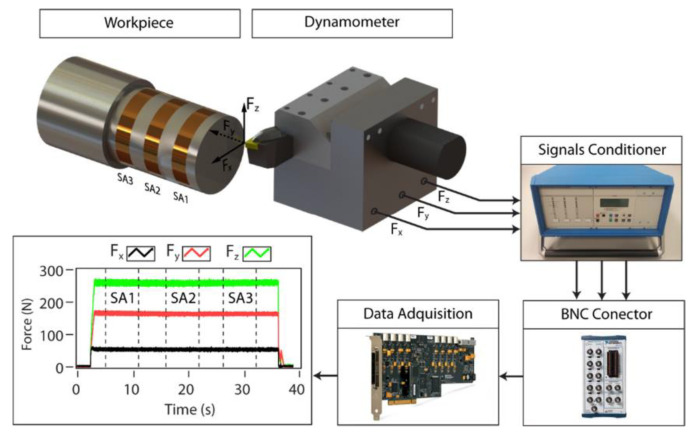
An example for cutting force based indirect tool condition monitoring system [103].

At the beginning of the machining, the cutting tool is sharp [99]. With the effect of high pressure and temperature, material loss occurs, and the contact conditions between tool and workpiece change in the progressive passes of machining [100]. The sharpness of the cutting tool reduces and the cutting process becomes difficult continuously [101]. Inevitably, there is need for higher CF to remove chip from material surface under same cutting conditions [102]. This situation refers to a relationship between TW and CF in theory. Besides, with the increased area between tool and workpiece as a result of TW, the same pressure generates more CF. Researchers intended to use the direct proportion between these two-process variable, CF measurements performed via several methods in the past. An example for cutting force based TCMS is given in Figure 4.

It is suitable to measure the three components of cutting forces during turning operation since each of them can be related to different kinds of TW. Different types and mechanisms of TW can progress on flank face or rake face depend on the load factors [4]. In addition, the cutting forces were generally measured in three dimensions, namely, tangential, axial and radial axes. For a clear definition between TW and CF components, it is necessary to investigate for each type of TW separately.

Kuntoğlu and Sağlam [5] performed an experimental study to investigate the sensor features and to find the relationship between CF components and FW in turning of AISI 5140. It was stated that tangential and axial cutting forces were highly reliable on monitoring of FW. Aslan [36] executed optimization and analysis of CF components during turning of AISI 5140 steel. The prediction accuracy and total percent contribution of cutting parameters were found lower for radial CF than tangential and axial cutting forces. Davim and Baptista [38] intended to find relationship between TW and cutting forces in machining silicon carbide reinforced aluminum. According to them, FW was the predominant TW, and three components of cutting forces demonstrated an increasing trend with FW. Moreover, axial and radial cutting forces were found more sensitive to FW. Suarez et al. [45] committed an investigation on turning of a nickel based alloy Haynes 282 to relate cutting force and TW. They reported that there was no visible relation between cutting forces and TW. Remedna and Rigal [42] investigated the evolution of tangential CF and TW during time in hard turning of an alloyed steel. It was observed that because of the mechanical contact between cutting tool-workpiece and machine tool cutting tool causes increasing in CF and changes its direction. Oraby and Hayhurst [41] demonstrated that tool force ratios provide more sensitive information using non-linear regression model for prediction of TW and tool life. They reported that force ratios are more accurate than absolute values of forces in predicting TW and tool life. El Hakim et al. [39] showed that in hard turning of high alloy steel with secondary hardening operation, CF and FW represented increasing trend simultaneously. Kuntoğlu and Sağlam [4] observed that tangential CF signal and FW increases with pass number in dry turning of AISI 1050 steel. Scheffer et al. [43] developed a system based on monitoring of FW in hard turning using three component dynamometer. All CF components demonstrated increasing behavior with increasing TW. Özel and Nadgir [35] developed a model based on CFs during hard turning of H-13 steel. According to results, it was possible to predict the FW for various cutting conditions. Sikdar and Chen [44] tried to correlate FW and component forces in turning of AISI 4340 steel. It was reported that tangential CF had the largest value pursued by axial and radial forces, respectively. It was seen that with increasing FW area, all components of CFs begin to increase. Kene and Choudhury [40] carried out an experimental investigation based on hard turning of EN24 steel for sensor fusion using CF alongside SR and vibration. Tool FW was predicted using different sensor signals and fusion approach provided better results than single sensor. Brinksmeier et al. [37] focused on measuring CFs and TW in high speed diamond turning, and it was found that both CFs and FW demonstrated increasing trend in time. Wu et al. [46] investigated micro groove tools for improving TW resistance in turning of AISI304. The authors researched FW and CF components and found that they indicated both forces and FW increase during time.

In references [4,5,35,36,37,38,39,40,41,42,43,44,45,46], a general opinion about the CFs is the increase in time with TW, shows sudden increase before tool breakage and decrease after tool breakage. Moreover, it can be stated that there is a consensus for dynamometer; this sensor is necessary for monitoring tool condition safely, reliably and accurately providing online information about tool life, TW condition and possible tool breakage.

**Advantages and disadvantages:** Dynamometer is a favorable sensor for this purpose due to its sensitivity and high reliability on measuring cutting forces. Dynamometer based CF measurement seems very popular and proper for reliable applications. Since the sensor is situated under the cutting zone, even small load changes can be detected. Although having serious investment cost, dynamometer was the most preferred sensor in the last 20 years in indirect TCMS. Dynamometer is the first choice for researches in terms of using sensor-based systems in turning which exhibits the reliability of this sensor in developing industry. On the other hand, the integration of the sensor is a challenging issue on occasion since its connection needs to be arranged considering the carriage and tool holder mechanism. In the scope of this study, FW related dynamometer usage is about 19.6% of all the presented papers in the last 20 years. The preference rate is high compared with the other indirect sensors which indicate the success of this sensor. For the future prospects, dynamometer integrated to machine tools initially is needed considering the importance of this sensor mentioned before.

### 3.2. Acoustic Emission

**Figure 5:**
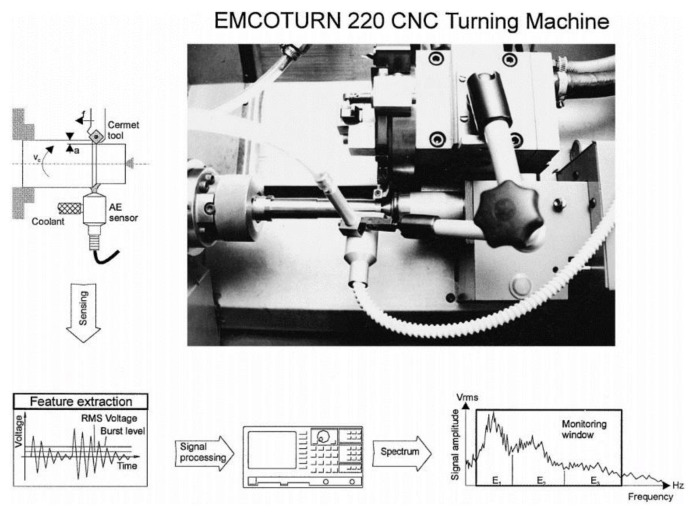
An example of acoustic emission based indirect tool condition monitoring system [107].

Acoustic means the science of sound including the vibration and noise which researches the propagation of their waves in solid, liquid or gas mediums. However, AE states the radiation of strain waves in a material subjected to an external load. This exposure leads to deformation of material in different levels such as degradation, breakage or wear. In other words, AE can be defined as the energy release that takes place in the material in micro scale which is deserted from other types of energies for example vibration, force and sound [6]. In machining and especially during turning, continuous contact among the cutting tool and workpiece produces a variety of shaped chips which causes different types of wear on the cutting tool as a result of plastic deformation [4]. The interaction between cutting tool, workpiece and chip appears as the main source of AE which end up with the friction, TW and tool breakage, deformation at contact zones, chip collision, chip breakage and chip tangling [104]. Among these effects, tool and chip breakage and some of the TW types, namely, chipping or built up edge, generate high amplitude AE signals, more commonly known as AE burst signals [84]. On the other hand, continuous type AE signals which possess low amplitude are generated with chip removing, TW and chip tangling. The characterization of AE signals provides information about machining mechanism and ensuing events in the cutting area throughout metal removing. Several AE signal features were applied in the past to distinguish worn and undamaged cutting tool [105]. That is why AE applications became the most popular technological approach in monitoring turning operations among the sensor systems in the last two decades compared to earlier. In Figure 5, an AE based TCMS is demonstrated.

The prominent motive of using the AE method is its capability of the sense the oncoming events and provides opportunity to take precautions for unexpected developments [106]. A similar approach was utilized in detecting earthquakes as it can be applied to determine the magnitude, duration and center of the earthquake. In this manner, AE method can be used as an early warning system especially in preventing failures which can be useful in practice for reducing cost during manufacturing processes. As a result, AE sensor is a very significant tool for the improvement of tool life, sustainability of machining process and administration of metal cutting operation [107].

In addition to providing information about different components such as cutting tool, workpiece and produced chip, easy implementation of the AE sensor brings esteemed advantage [108]. Since the closeness of a sensor to the cutting area increases the precision of the collected signals, small-dimensioned AE sensor possesses another advantage. The adaptation of the sensor can be performed with screw-nut connection which provides great convenience in determining the location.

Considering its benefits, wide range of usage of the AE sensors is an expected result with the reflection capability of the healthiness of the cutting tool. Kuntoğlu and Sağlam [4] compared the AE and tangential CF signals at the time of tool breakage during turning AISI 1050. They found that tangential CF provides significant information at the breakage moment; however, AE signals detect chipping before the failure and give considerable clue about the oncoming event. Bhuiyan et al. [48] applied AE sensor into turning operation during machining ASSAB 705 steel to research the frequency ranges of TW and plastic deformation of workpiece material. It was reported that the separation of the frequency of TW and plastic deformation was difficult during turning. According to them, when the material removal rate or TW demonstrates increasing trend, AE signal also increases. Maia et al. [51] proposed an innovative approach using AE sensor during turning of AISI 4340 steel to determine the tool life and wear mechanisms. According to the results, newly developed method of power spectral density used for processing AE signal provides notable information about TW rate and tool life. Moreover, the proposed method was found as sensitive and effective in detection of wear mechanisms. Kuntoğlu and Sağlam [5] indicated that AE sensor signal is one of the major indicator of tool breakage alongside vibration and CF signals. Scheffer et al. [43] proposed a TCMS based on monitoring of FW in hard turning using AE sensor. AE signals indicated decreasing trend during the normal worn phase and increasing behavior because of high temperatures as FW increases. Bhuiyan et al. [47] proposed an AE sensor based monitoring system in turning of ASSAB-705 steel as it represented the chip formation on tool state. It was found that TW can be detected as decreasing with chip breakage and verification was performed. Neslusan et al. [52] detected tool breakage successfully during hard turning of 100Cr6 steel using different AE sensor signal parameters. Chethan et al. [49] focused on optimization of machining parameters and TW in turning of Nimonic-75. With the help of machine vision, AE sensor signal parameters successfully measured the wear area. Chiou and Liang [50] carried out an analysis on AE signals to detect chatter vibration with TW effect in turning of 6061-T6 aluminum workpiece material. It was demonstrated that AE root mean square signals effectively distinguish fresh and worn tools, confirming chatter vibrations. Wang et al. [53] used AE sensor for TW evaluation and categorized burst and continuous signals by their triggering mechanisms in turning of Inconel 182 material. According to results, tool FW can be detected via burst signal which occurred from fracture and plastic deformation.

References [4,5,43,47,48,49,50,51,52,53] indicated that AE sensor signals have great importance on detecting tool FW and possible tool breakage with the help of various signal features identified theoretically. Being a non-destructive method and easily implemented structure for monitoring area, having various signal features for characterization of different phenomena, namely, plastic deformation, TW, chip production and tool breakage, AE sensors are heavily preferred in TCMS during turning operation.

**Advantages and disadvantages:** The characterization of AE signals provides information about machining mechanism and ensuing events at the cutting area throughout metal removing. Minor and major changes during the cutting operation can be precisely sensed via AE sensor when it is positioned in the right way. This brings a clear advantage to distinguish the events as normal ones maintain the cutting and abnormal ones endanger the health of cutting. According to utilization, the sensing scale of AE sensor should be clarified. In a very large scale, some data losses lead to late detection or missing which can end up with tool failure or substantial damages. Especially for the newly developed materials in industry, unknown plastic deformation mechanism and possible tool breakage scenarios push the researchers to use the AE sensor. It is needed to observe the fluctuating changes occurring in the material to better understand the behavior of cutting tool and workpiece during cutting operation. AE sensors have been preferred in the last 20 years covering the FW detection with the rate of 16.3%. Considering the increasing effect of the richness of the information on detection of tool wear and tool breakage, it is reasonable to select this sensor in wide scale. It was observed on the previous study from author [5] that AE sensor had its most effective signal feature among the other sensor signals in detecting FW with the high success rate of 74%. AE sensor provides clearly a positive contribution to the perspective of Industry 4.0 with its capability for new generation materials if the high costs can be compensated.

### 3.3. Vibration

**Figure 6:**
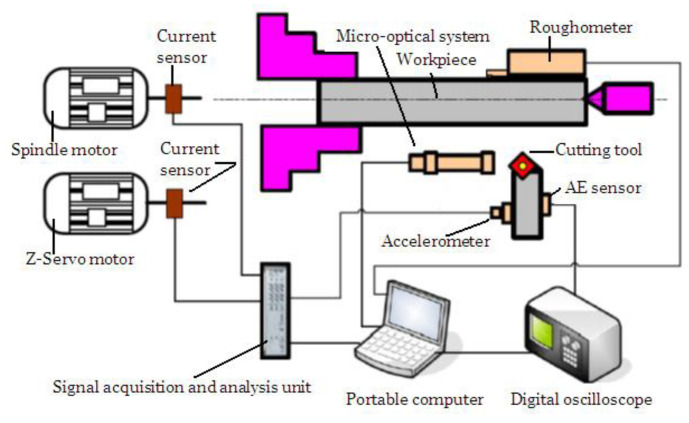
An example for vibration, current and surface roughness based indirect tool condition monitoring system [112].

In order to obtain the desired results from the machining process, to keep the process variables under control and to avoid undesirable results, the necessary conditions must be provided to continue the process in a stable manner [31]. In this regard, vibrations occur because of the lack of rigidity in the machine, errors in the workpiece and cutting tool connecting procedure or depending on the altering cutting conditions during the machining process [36]. Vibration is an undesirable condition that adversely affects the machining process. In addition, a vibration occurs in the system continuously as the CF occurring during machining depends on many factors such as distance between cutting edges, position angle, workpiece geometry, spindle deflection, depth of cut, chip width, feed rate, cutting speed and especially the TW [5]. Considering the literature survey, it has been observed that many diverse techniques are used to monitor TW. These methods are expressed under two main headings, directly and indirectly. The first technique is to measure TW directly on the tool by finishing the material removal process. In the second technique, different parameters associated with TW such as motor current, force and moment, vibration, acoustic and SR values are measured with appropriate test instruments. These measurements are evaluated using various analysis and estimation methods, and the amount of wear in different time intervals can be calculated [109]. Estimation methods are based on modern and topical approaches such as fuzzy logic, artificial neural networks, genetic algorithms, as well as statistical methods [6]. Although several studies on process modeling with the help of artificial intelligence have been conducted by various researchers, a practical method has not yet been developed to monitor TW in the industry. A variety of researches are still carrying on monitoring tool condition, controlling machining processes and ensuring their optimization and prediction. In Figure 6, vibration based TCMS is shown in addition to current and surface roughness measurement.

In machining, vibration is considered one of the variables that best reflect the TCM process. Factors such as ease of application and no need to make any arrangements to the machine tool or workpiece fixture are among the most important advantages that distinguish vibration measurement from the aforementioned parameters. Therefore, vibration monitoring procedures are widely used to diagnose and predict part accuracy, surface texture, and especially tool condition. Vibrations occurring during machining are classified into two classes as vibrations dependent on the cutting process and vibrations independent of the cutting process [110]. The type of vibration that occurs as a result of TW during machining is called vibrations that are dependent on the cutting process. Therefore, monitoring TW requires recording and monitoring vibrations that depend on the cutting process [4]. TW increases during cutting and these increases lead to an increment in vibration amplitude [5].

Aghdam et al. [54] examined that the relationship between the vibration characteristics of the tool and holder assembly and the major flank wear of the tool in a turning operation. They measured the tool acceleration signals generated during cutting and then determined wear-sensitive properties according to the dynamics of the tool/holder system, which manifested itself at natural frequencies. They determined the recorded signals using the autoregressive moving average model. According to the analysis of the experimental results, they found that in the speedup phase of wear, a vibration mode changed from the second bending mode in the main cutting direction to the first bending mode in the feed direction and simultaneously the autoregressive moving average distance reached a minimum value. They also stated that the analysis they conducted provided a reliable algorithm for TW prediction, as it directly originated from the tool holder and system natural frequencies and interpreted the physical behavior of the system in connection with TW.

Dimla [111] conducted a study on evaluating the TW monitoring procedure in a metal turning process utilizing vibration characteristics. Considering analysis results, they reported that the properties of the vibration signals were a useful feature for monitoring cutting TW and determining the wear quality. Sick [33] examined diverse vibration monitoring methods which are used to estimate flank wear and suggested that regularization process parameters at the digital preprocessing stage could improve the correctness of the results. Scheffer et al. [43] presented a system based on TCMS for FW in hard turning using three component accelerometer. Vibration energy indicated increasing curve in respect of TW. Kene and Choudhury [40] utilized vibration signals to predict FW in hard turning of EN24 steel. On the previous studies from Aslan [36] that vibration can be supportive to CF signals in monitoring of FW during turning of AISI 5140, Kuntoğlu and Sağlam [5] stated that vibration was not very effective on detecting FW compared to AE and temperature signals in turning of AISI 5140 steel. Alonso and Salgado [55] analyzed vibration signals for detection of TW in turning of C45 steel and reported that the proposed method was very effective in TCM. Ghani and Choudhury [57] presented a study during turning of cast iron, and they stated that at low depth of cut values, with the increase of FW, vibration remained constant. Chiou and Liang [50] analyzed AE signals for chatter vibration to detect TW in turning of 6061-T6 aluminum, and it was found that this approach was very sensitive in monitoring of TW. Kataoka and Shamoto [58] revealed that flank face interfered with the workpiece material with increasing of vibration, which leads to increasing of abrasive wear in turning of AISI 1045 steel. Prasad and Babu [59] tried to correlate vibration and TW in turning of AISI 4140 steel, and the authors indicated that there was a close relationship between TW and vibration.

In the references [5,33,36,40,43,50,54,55,57,58,59,111], it was stated that there was a correlation between FW and vibration signals; however, the reflection capability of vibration signals might not be the solution on its own in the TCMS.

**Advantages and disadvantages:** One of the distinctive advantages of this sensor is to provide information about one of the main problems in machining. Vibration ruptures the nature of machining by changing the pre-determined cutting tool-workpiece contact conditions which further damages the tool structure and have devastating influence on workpiece. Thus, integration of an accelerometer helps to standardize the machining condition as stable during continuous vibration and unstable when excessive vibration occurs. Industrially applicable accelerometers can be easily mounted on various points of the machine tool for measuring the stiffness, in addition. Especially for finish turning operations and internal turning, vibration becomes a very challenging issue. With the increasing demand from customers, high accuracy products will be the most important thing for a company in terms of the reputation in market. The given applications based on accelerometer and vibration measurement are about 19.6% among all indirect monitoring systems including the studies in the perspective of this paper. A considerably high amount of usage about this sensor is found which exposes the validity and reliability of the effect of vibration on FW. Considering its high value contributions to a manufacturing chain, the investment cost of this sensor can be ignored in the objection of Industry 4.0.

### 3.4. Temperature

In all metal cutting processes, the main aim is to overcome the shear strength of workpiece material with the cutting tools. This overcoming process generates a large amount of heat in the workpiece. Plastic deformation of the workpiece is facilitated by the increase in temperature in the workpiece, since high temperatures decrease the yield strength of workpiece, and this decrease causes an increase in the capability of plastic deformation of workpiece. Metal removal process occurs in the tool-workpiece area as a result of plastic deformation.

**Figure 7:**
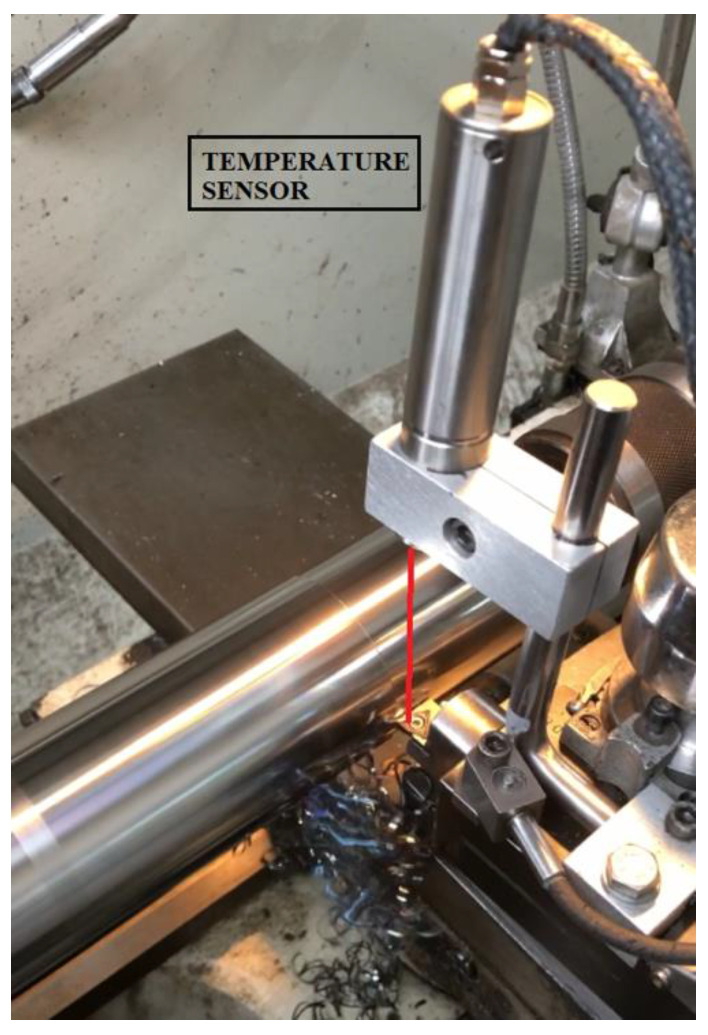
An example for temperature based indirect tool condition monitoring system.

In machining processes, most of the energy transferred to the machine tool is converted into heat energy. The resulting temperatures are dissipated between the tool and the workpiece and the chips. While the cutting tool and workpiece are in contact, chips are removed from the workpiece by means of pressure and temperature. Whilst presence of temperature increases the plastic deformation ability of workpiece, at the same time, it makes monitoring of tool condition difficult. Figure 7 indicates temperature based TCMS as an example.

El Hakim et al. [39] indicated that in hard turning of high alloy steel with secondary hardening operation, temperature and flank TW demonstrated an increasing trend. Increasing cutting speed triggers the cutting temperature, and it leads to increase in TW. Increasing cutting speed and feed rate parameters were two of the most effective parameters which control the increase in temperature. Yıldırım et al. [66] reported that increasing feed rate (0.1, 0.125, 0.15 mm/rev) reduced to tool life by 32.73%, 29.89% and 38.01%. This tool life decrease is a result of high temperature between tool and workpiece. When the dry machining and nano-minimum quantity lubrication system were compared, it could be seen that the tool life could be increased by 105%. If the nano minimum quantity lubrication system was applied at the ratio of 1 vol%, the temperature could be decreased by approximately 30%. Another study was reported that high temperature levels increased the adhesive and diffusive TW, and they should be controlled by a lubrication strategy based on the machining process [65]. Sasahara et al. [64] presented a study including the effect of the temperature on the tool life. The workpiece material was SUS304 stainless steel, and two different machining conditions, which were dry and minimum quantity lubrication, were investigated. When the cutting speeds were selected as 100, 300, 500 m/min, the temperatures were measured as 550, 850, 1000 °C respectively in the dry machining condition. However, the temperature values decreased after the minimum quantity lubrication process at the ratio of 10%, 5%, 8% reduction of temperature depending on the lubrication system extending the tool life. Das and Chapagain [61] presented a study that investigated cutting parameters on temperature in turning of aluminum matrix composites. While the feed rates were 0.05, 0.1, 0.16 and 0.2, temperatures increasing from 36.60 to 71.67 °C depend on the feed rate increase. When it came to cutting speed (40, 106, 169, 206), the temperature increased from 36.60 to 83.50 °C. While depth of cut was also an effective parameter on the temperature increase, it was determined that the most effective parameter was cutting speed. Effects of the cutting speed on the temperature in in dry hard turning of Inconel 718 were studied by Zhao and Liu [68]. It was reported that temperature shows an increasing trend with the increase of cutting speed. The temperature values can decline up to 30 °C by means of coating of cutting tools. Özbek and Saruhan [63] presented a study related to effects of cutting zone temperature on TW. It was reported that both cutting tool coating and cutting speed have a great impact on cutting zone temperature, and this temperature can be reduced by minimum quantity lubrication system, approximately 100 °C. One of the possible explanations of low temperature owing to minimum quantity lubrication system was that minimum quantity lubrication system moved away the chips from the surface with high pressure. Moreover, it was indicated that this phenomenon can be observed via thermal images.

There were some studies in comparison with the studies which reported the cutting speed was the most effective parameters on temperature increase. Kuntoğlu et al. [31] reported a study that depth of cut is the most effective parameter on the increase of cutting zone temperature. The reason was that high depth of cut values leads to large amount of metal chip removal, and the cutting tool undergoes a more restrictive force. Due to the chip removal and the restrictive forces, the workpiece was under the shear effect. In another study which supported these evaluations belonging to [48], the effect of depth of cut on temperature and TW was investigated, and they reported that depth of cut was the dominant cutting parameter rather than cutting speed and feed rate. There were other studies [62,67] which indicated the most important parameters were depth of cut on the temperature increase and TW progression.

In [31,39,48,61,62,63,64,65,66,67,68], it was reported that a correlation between cutting temperature and TW was found, mostly triggered by cutting conditions and cutting speed. Temperature based measurement provided significant contribution for detecting TW progress and tool life in the field of TCMS. These articles had in common that the information about cutting temperature could be very important for accurate detection of tool condition.

**Advantages and disadvantages:** Temperature measurement can be achieved with inexpensive sensors; it is one of the principal advantages. Since the most part of the heat dissipated to removed chips during machining, and the main aim is to measure the temperature on cutting tool, collecting signals from cutting area becomes almost impossible while machining continues. It is possible to measure the temperature of chips and then learn the approximate value of cutting tool and workpiece using transition equations. This may lead to an error to obtain the exact value which becomes a disadvantage when working high range of temperatures. On the previous study published by author [5], it was stated that tool temperature sensor signals are quite effective on detecting tool FW (74%). On the other hand, the papers examined in the scope of this study tend to use temperature measurement for monitoring of FW, which is close to usually preferred cutting force measurement proportionately (18%).

### 3.5. Motor Current

**Figure 8:**
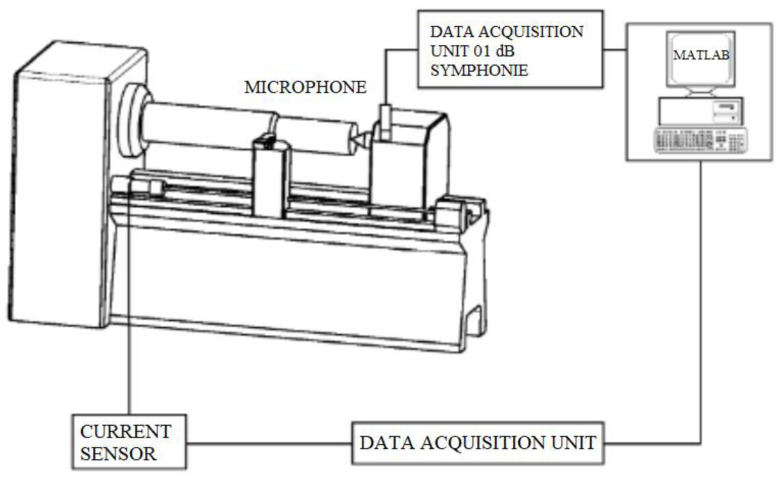
An example for sound based indirect tool condition monitoring system [73].

In situ control of turning process is a difficult task due to the stochastic nature of TW. This leads to great challenge in terms of achieving optimum conditions [41]. Time-dependent wear of cutting tool changes the predetermined cutting conditions and affects process variables considerably [113]. Motor power and motor current are the main sources for metal cutting and are associated with the changes at the cutting area especially for the TW. In theory, it is expected that with the increase of cutting TW, CFs demonstrate enhancing behavior, and this directly affects the cutting power and current [29]. However, the utilization of current sensor in TCMS is very little compared to the aforementioned sensors, i.e., force, AE, vibration and temperature. In the author’s previous study, current sensor was found as the third effective signal on FW among the seven sensor signals [5]. Nevertheless, it was observed that the sensor signal was ineffective to sense the approaching tool breakage. In Figure 6 and Figure 8, current based TCMS are shown.

A current based TCMS is hard to find in open literature for turning, especially its correlation with TW [24]. Some attempts about current measurement during milling and drilling were undertaken, yet none of them related to TW [114,115,116]. Szecsi [74] used DC motor based TCMS to evaluate TW, and according to the author, armature current of the main motor can be informative to determine the condition of tool during CNC turning. Kuntoğlu and Sağlam [5] established a TCMS for FW and tool breakage monitoring, and current sensor was found to be successful after AE and temperature sensors on monitoring of FW in turning of AISI 5140. Kuntoğlu et al. [6] optimized the cutting conditions using sensor couples for FW among the five different sensors. Current sensor was successful but, however, not the best option. Salgado and Alonso [73] presented an approach based on using current signals for online TW monitoring during turning of AISI 1040. They estimated axial CF using current signals for further evaluation of FW within high success rate. Yip and To [75] integrated eddy current damping to reduce vibration for turning of titanium alloy with the purpose of enhancement of tool life.

Even the current sensor indicated promising results for multiple optimization, TCM and FW and tool breakage monitoring in the past studies [5,6,73,74,75]; there is a long way to go in better understanding the structure of this sensor during turning operation.

**Advantages and disadvantages:** Principal requirement for a sensor is being close to the cutting area because of the increased sensitivity. The main disadvantage of the current measurement is its connection to cutting area is that it is performed with current transfer cable, which transmits energy to machine tool. Different from the other sensors in indirect TCMS, current sensor is situated at a distance, and this leads to loss of precision. Due to the mentioned drawbacks of this sensor, it has been preferred rarely (8.1%) in the past for progressive FW detection in turning. In addition, current measurement is important as one of the first sensors used in TCMS in the 1980s. This makes this sensor the earliest one in the indirect TCMS methods, which is highly possible, were used by many of the researchers. Eventually, it is concluded that current measurement can assist and support more accurate sensor signals, namely, force or AE in detection of tool breakage.

### 3.6. Sound

It is basically generated sound that exists in all machining operations as a result of high speeds for cutting of hard materials. In milling, intermittent cutting produces also discontinuous sound due to consecutive entry and exit of cutting tool into workpiece material. However, in turning, continuous contact between tool and workpiece produces different shaped chips, depending on cutting conditions and material properties. Besides, a new cutting tool is effective on metal removing compared to a worn cutting tool. This makes difficult to cut, and scraping starts instead of cutting as a result of the changing cutting tool geometry. An impaired cutting mechanism affects the resultant sound, and this can be an information source for the condition of TW [24]. Sound measurement is fundamentally done with microphone; however, sound signals have some drawbacks. The separation of frequency range of sounds originated from chip formation, TW and breakage, machine tool, and vibration should be performed accurately to detect the desired quality characteristic. For this reason, this sensor is preferred over the two other sensors.

Sound sensor was preferred in [73] for reliable and faster decision making in addition to acceptable cost-performance ratio. Favorable estimation error for TW was obtained according to results. Lu and Kannatey-Asibu [71] performed sound sensor based experimental work to monitor FW, and they reported that it can be useful and execute the ability of operator. Mannan et al. [72] applied sound sensor for analysis of TW during turning of AISI 4340 steel. According to authors, the proposed method can successfully distinguish sharp tool from dull tool. On another study from Abu-Zahra ve Gang [69], it was demonstrated that because of the acoustic behavior of ultrasonic waves, TW can be monitored successfully by this method. Kopac ve Sali [70] carried out experimental research to predict TW using online TCMS during turning of Ck15 and obtained results showed that increasing TW was correlated with the increase in amplitude of sound.

Considering References [69,70,71,72,73], it can be concluded that sound signals seem very effective on detection of FW according to the authors’ reports. Low cost and easy to implement structure of this sensor enable to use it in TCMS in turning operations for different types of materials.

**Advantages and disadvantages:** A microphone resembles an operator ear somehow, which was utilized from the beginning of the machining for health monitoring. Considering sustained success sound-based monitoring provides quite important benefits. However, compared with AE sensor, reaction time is very long, which makes the microphone less reliable. Because of the more profound and complex structure of machining the moment of an event such as breakage can be detected later. This makes the method not a first choice but a supportive one for confirmation of the other sensorial data. In the past, a few researchers integrated the sound sensor into a turning operation with the purpose of FW monitoring according to findings (8.1%). The peculiar type of the microphone in machining leads to neglect of this sensor; however, the similarity between AE can provide sensitive detection of tool breakage.

### 3.7. Tool Flank Wear

**Figure 9:**
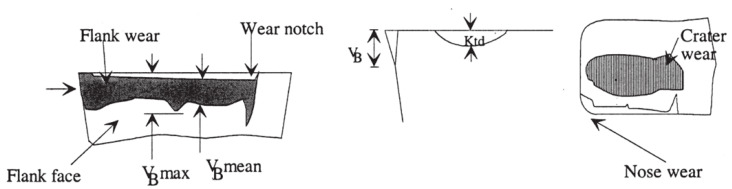
Types of crater wear and flank wear to standard ISO 3685:1993 [99,117].

TW generally occurs in the two main areas of the cutting tool (chip surface and side surface of the cutting tool). Therefore, TW is generally divided into crater wear and FW (Figure 9) [99]. The evaluation of tool wear is applied according to standard ISO 3685 [117].

FW is the type of wear caused by the friction that occurs between the insert clearance angle of the cutting tool and the new surface of the part corresponding to this angle. The width of the FW is indicated by V_B_. Mechanical, chemical, diffusive seem to be the major load factors affecting the FW [118,119].

FW develops on the flank face of the cutting tool as a result of several parameters. Too high cutting speed, plastic deformation resultant of too high cutting temperatures and edge chipping caused by excessive load on the cutting tool are the main factors on FW [119].

In the studies made on AISI 4340 [120,121], AISI 4140 [122] and AISI D6 [123] steels, it has been revealed that the most common wear type is FW and crater wear. It is also stated that the most common wear mechanism for these wear types is abrasive wear. In the study using AISI H11 [124], AISI D3 [125], AISI 4340 [121] and AISI D6 [123] steels, the most effective cutting parameters for wear were cutting time, cutting speed, feed, and depth of cut. In addition to these parameters, the relationship between tool nose radius, insert edge chamfer, cutting temperature, coating layer and lubrication with TW was also investigated [126,127,128,129,130].

In turning, it is important that the tool continues cutting operation with high performance for optimum tool life [131]. Although there has been a recent trend of transition to hard material turning processes in terms of surface quality, one of the main problems in both turning these materials and turning normal materials has been rapid TW. The use of cutting tool inserts such as cubic boron nitride, polycrystalline diamond and ceramic was intended to prevent rapid TW; however, high tool costs were observed. Recently, however, the most suitable cutting conditions have been developed to ensure minimum TW and minimum production cost [132,133,134,135]. In addition, modeling and optimization techniques such as artificial neural network, fuzzy logic, multiple regression, response surface methodology, variance analysis, and Taguchi method were used in the development of these conditions [36].

Kuntoğlu and Sağlam [5] performed turning process on AISI 5140 steel and they integrated five different sensors on the lathe to examine the effects of CFs, vibration, AE, temperature and current measurements on FW. According to the results, temperature and AE signals are 74% effective on FW. Çetindağ et al. [136] investigated the effect of AISI 52100 steel on FW by performing turning process with conventional and wiper cubic boron nitride inserts. According to results, they observed that the wiper cubic boron nitride insert significantly reduced TW. Dudzik et al. [137], during the turning process on 304L stainless steel, observed by comparing the AE signals and CFs to predict TW, that both main variables are important in predicting TW. Twardowski et al. observed the TW of 100Cr6 steel with the turning process in their study. In the study, in which shear forces and mechanical vibrations were observed, FW was estimated with artificial neural network. According to the authors [138], the physical measurements of the estimation with artificial neural network are appropriate. Aouici et al. [139] compared the FW for the wiper ceramic insert with the traditional ceramic insert in dry hard turning of AISI 4140 steel. Analysis of variance was carried out in this study, and cutting speed, feed rate and depth of cut were also used as variables. According to the experimental results, it was shown that the wiper ceramic insert performs better than traditional ceramic insert in terms of FW.

**Advantages and disadvantages:** Since the tool wear inevitably develops on the cutting insert, it should be kept under control to understand the underlying mechanism for avoiding excessive levels. In this direction, one of the main advantages of it is that online monitoring of tool wear is feasible with indirect sensors without stopping the operation. Due to FW increases on the main cutting edge and proper for quantifying with standards, its detection offers vital information about the remaining useful life of the cutting tool. However, the sophistication because of the numerous cutting parameters and variables aggravates accurate estimation of tool wear on occasion. In order to validate the wear progression rate, this is necessary, which leads to high investment cost for reliable and sustainable manufacturing.

### 3.8. Surface Roughness

In engineering applications, the desired surface shapes in part are expressed as the nominal surface. The surface texture consists in repeated deviations from the nominal surface of the part, and these deviations are the following:

1. Waviness (Irregularities with measurement ranges greater than surface roughness sampling distance);
2. Defects (scratches, cracks, stress concentration and alignment errors);
3. SR (average of vertical deviations of a certain distance of a surface that has undergone a certain treatment).

These are expressed by their properties. The average of vertical deviations at a certain distance of a surface that has undergone a certain treatment is called SR. Today, the method of average SR is frequently used as a method of determining SR. This method uses an arithmetic mean based on the absolute value of deviations, and this method can be expressed by Formula (1) [119].

(1) $$R_{a}=\overset{L_{m}}{\underset{0}{\int }}\frac{\left|y\right|}{L_{m}}dx$$

In this formula, it is defined as *R_a_* = average arithmetic SR, *L_m_* = distance measured, *y* = deviation from nominal surface. Although there are many methods to measure SR values, needle point devices, and devices using optical methods (laser, light diffusion, etc.) are among the most used methods. Besides, recently, there were techniques in which 3D graphics of the surface were created to detect SR [140].

Considering the technological developments in recent years, it can be seen that excellence was aimed in terms of product quality. In Figure 6, a surface-roughness-based TCMS is shown. The quality expectation is very high, especially in areas such as automotive and aircraft industry, SR is the only criteria that provide this excellence [141]. SR is an important output, especially for parts manufactured in the machining process. The purpose of machining is not only to shape the part but also to realize the correct process in terms of surface quality [142,143]. SR is a very important process variable in turning as being an ultimate aim. Cutting tool radius, feed and cutting speed can be shown among the factors that significantly affect the SR in turning. Aside from the material of the workpiece, vibration on the machine tool also affects the SR, and these factors were statistically significant on the SR [140].

The parameters affecting the SR can be evaluated in two main groups as dependent and independent variables. While parameters such as AE, vibration, temperature, CFs and TW are dependent variables; feed rate, depth of cut, part material and insert are shown as the parameters that affect the SR independently [144,145,146,147]. With these parameters, the aims of studies on SR have been to minimize SR [148].

In recent years, SR estimation and optimization of these values have become very important for the industry. In steels, AISI 4140 [122], ADI (grade 3) [149], AISI 1014 [150], AISI 1060 [62], AISI 1040 [151], AISI 316Ti [152] and AISI 52100 [153], optimum SR values were estimated by determining the interaction of optimization methods and independent variables for SR using Taguchi method, response surface method, variance analysis and regression models. Moreover, with these values, information about SR values can be obtained by using the ant colony algorithms, fuzzy logic and artificial neural network. By creating an artificial neural model after turning, this network is trained, and after the network training, intermediate values can be estimated with this model and gain from time and cost criteria [14,144].

Kuntoglu et al. [31] performed a turning process on AISI 5140 steel and estimated the SR with the response surface methodology. For this estimation, response surface method-based quadratic regression models were obtained, and they determined the optimum cutting conditions by different cutting speeds, feed rate, cutting edge angles, and axial-radial-tangential vibration inputs. In the study, they obtained high accuracy surface prediction values using these models. Besides, according to the results of the study, they determined that the input that affects the SR the most are the feed rate and then the axial vibration. Zhou et al. [154] created an algorithm based on the genetic-gradient boosting regression tree for the optimization of cutting parameters and SR estimation by turning AISI 304 stainless steel. They compared this model created with an optimized artificial neural network and support vector regression method. The developed algorithm based on genetic-gradient boosting regression tree is a better method than other prediction models used in the study with a root mean square error index of 0.087. Tokarev et al. [155] performed a turning process of 40X steel with an insert with a pile layer built up edge and analyzed the effect of built up edge on the SR as a result of turning by simulating a mathematical model. As a result of the examination, they emphasized that the data obtained from this model both experimentally and theoretically were satisfactory. They also stated that the adhesive properties of the piece material have a significant effect on SR. Vasanth et al. [156] developed regression models and artificial neural network based on CF, cutting temperature, TW, and tool vibration to estimate the SR value after turning using hardened SS 410 steel. They stated that artificial neural network gives more accurate results compared to the depression model, and they get more accurate results with more input for the neural network.

### 3.9. Image Processing

**Figure 10:**
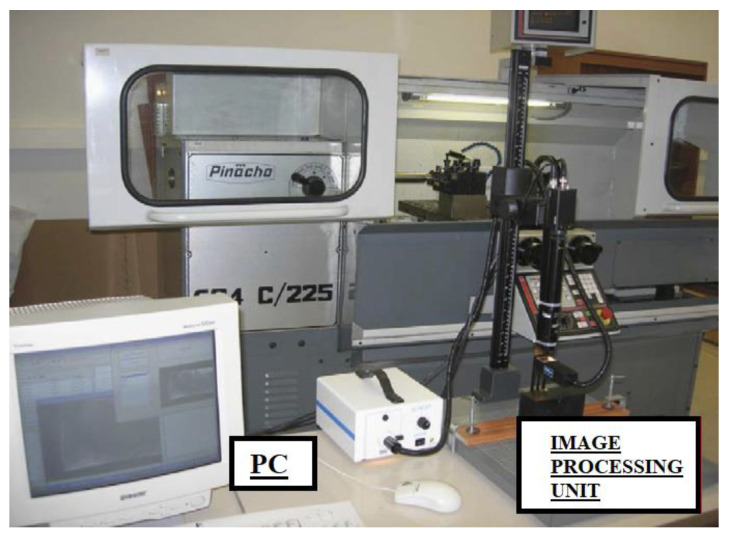
An example for image processing based tool condition monitoring system [77].

Image processing is a pattern recognition process that works with a strategy based on measurement of image textures in a variety of application areas such as statistical data analysis, machine learning and signal processing [78]. These systems simply need a camera to divide the image into segments for an intense analysis since there are repetitive patterns on an image [157]. The primary challenge for pattern recognition method is to segment a whole image and determine boundaries. After that, it is required to describe the characteristics of each region for further analysis and decision making. In the measurement of TW and FW in the concept of this paper, the worn region on a cutting tool needs to be distinguished from the unworn region. Once textures are identified as worn or unworn in a moment during the machining, the state of the wear level of cutting tool according to cutting time is described. In Figure 10, an image processing approach is shown.

Mannan et al. [72] used image processing technique for analysis of TW during turning of AISI 4340 steel under the cutting conditions where the tool reached catastrophic failure, and they stated that this approach was successful in identifying sharp, semi dull and dull cutting tools. Kerr et al. [78] used EN24T material to recognize the extent of FW via image processing technology. It was stated by the authors that, although this method had potential in evaluation of image for tool condition, there were still need some problems to overcome. Mikolajczyk et al. [79] adopted image processing and neural networks in turning of C45 steel for predicting tool life. This approach provided high error results for the excessive TW values; the accuracy of prediction was satisfying and proper for the industrial applications. Barreiro et al. [76] used digital image processing method to determine the tool life and obtain useful lifetime of a cutting tool. Their discovery provided reduced tool costs using a new wear criterion. Castejon et al. [77] proposed an online wear monitoring system with geometric descriptors from linear images. Their results indicated that the approach might classify wear as low, medium, high. Pfeifer and Wiegers [80] utilized machine vision system with adaptive illumination to optimize images for different usage and different types of cutting tools. Mikolajczyk et al. [81] utilized neural network approach using image analysis for tool wear and found the tool wear results within a very low error rate.

According to papers [72,76,77,78,79,80,81], it was advised that image processing based on machine vision, pattern recognition and image analysis methods, because of its usefulness in determining tool life, TW condition and possible tool breakage, might be very effective for cost optimization and productivity.

**Advantages and disadvantages:** Different from the mentioned sensor systems, image processing is applied after machining operation is completed. This situation brings a disadvantage since the prevention of tool breakage and excessive tool wear becomes difficult and almost impossible. However, when the machining stopped for different reasons, such as measuring of surface roughness, it is possible to employ pattern-recognition-based applications. This approach provides additional information about tool and can be beneficial for confirmation of the collected sensor data. It is noteworthy that well-structured software can predict the situation of the tool within high success rates, which improves the quality of machining by far. According to published papers in the field of monitoring of turning operation for FW, it was found that 9.8% of the studies preferred image processing techniques for that purpose. This can be explained by the incapability of these systems to intervene in the machining processes.

## 4. Data Acquisition and Signal Processing

**Figure 11:**
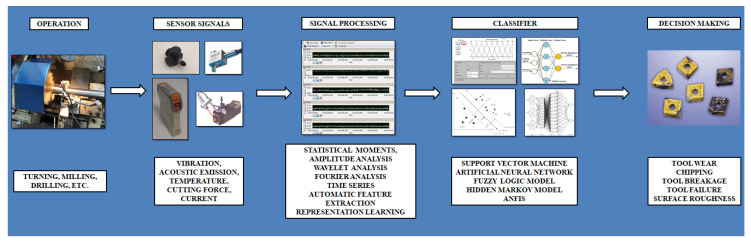
The whole procedure of monitoring of tool condition via TCMS.

After the experiments under specified cutting conditions, gathered data needs to be evaluated with proper methods, which give accurate information to operator or machine learning algorithm for final decision. In the signal processing phase, the researcher needs information about the analysis method ideally reflecting the signal features. Very popular signal processing methods to evaluate tool condition are outlined in this topic [158]. Seemingly, statistical moments, amplitude analysis, wavelet analysis, Fourier analysis, time series modeling, automatic feature extraction and representation learning are the best options in open literature for data acquisition and signal processing phase. It is desirable, during data acquisition and signal processing procedure, to extract proper features which correlate two variables without affecting noise factors. For deriving various features, different type of domains, namely, time [159], frequency [160], time-frequency [43] and statistical [161] ones, were selected in the literature. It was found that force signal dependent features were extracted in time domain. In this manner, sound, AE and vibration signals were extracted in frequency domain. Wavelet analysis is generally preferred to extract features for time-frequency domain since its capability to provide signals time and frequency simultaneously which enables changes signals from one series to two dimensional function [162,163]. Fourier analysis is generally preferred to extract signals using fast Fourier transform signals which have a principle for extracting frequency components of the handled data [161]. Statistical moments extract features in statistical domain using coefficients belonging to time series modeling, namely, auto regression, moving average, etc. [22]. The general approach is to calculate the distribution using variance, skew, kurtosis. Automatic feature extraction offers to determine several kinds of characteristics reflecting the system in the best way in terms of the investigated parameters using a variety of artificial intelligence methods [164]. Representation learning or feature learning provides capability to select specified features for the monitored process variable [165]. The previous couple of signal processing methods are accepted emerging technologies as the use of deep learning methods. The whole procedure of TCMS in monitoring of tool condition is summarized in Figure 11. It explains respectively the operation type, sensors and sensor signals, signal processing methods, data classifiers and decision-making phases.

## 5. Decision Making Methods

Gathered data from sensors through data acquisition systems was handled for final decision about the quality characteristic [113]. This process requires a second expertness in terms of the researcher who should know mechanical process and modeling well. This comes from the TCMS being a multi-disciplinary research area containing mechanical systems, sensors and computer software. In brief, processed signals should be categorized via classifiers, and this phase is generally performed with artificial intelligence approaches. The popular classifiers preferred in literature for decision making are listed in Figure 5. Since there were limited studies about the relationship between sensorial data and FW in the open literature, the general point of view in this topic is to summarize the state of the art of each decision-making method on available example/s. The most popular decision-making methods in this area are artificial neural network, fuzzy logic, hidden Markov model, support vector machine and adaptive network based fuzzy inference system.

### 5.1. Artificial Neural Network

**Figure 12:**
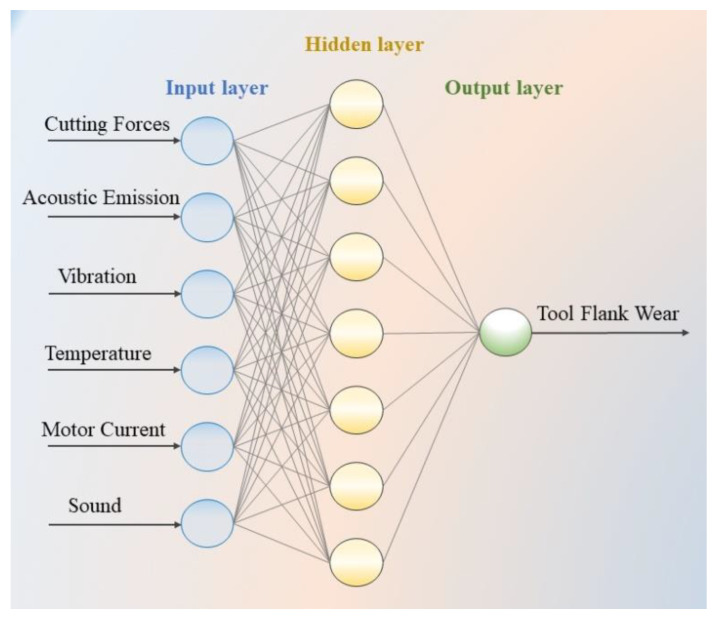
The structure of artificial neural network model.

One of the most popular decision-making methods is artificial neural network model inspired by the working mechanism of the human brain. The model contains three or more layers, each one set with number of neurons. The first and last layers indicate the input/s and output/s, respectively, and the neurons in these layers should be connected to each neuron in the hidden layers. One crucial point during modeling of neural network is the necessity to have experience in and knowledge about determining the number of hidden layers and neurons. However, depending on the inputs and outputs, the relationship between parameters, number of hidden layers and their neurons needs to be revised and constructed. The connection between each neuron in entire layers is provided with weights which require training for the best solution. After the training procedure, it is expected to obtain the value of the response parameter from the output layer. There are several factors affecting the performance of the neural network structure such as training time, transfer function, weights, learning function [166]. In addition, a number of deep learning methods such as convolutional networks and recurrent neural networks [164,165], accepted as the top performing models, have been applied recently. Recent advice in the field of health manufacturing indicates the importance of these state-of-art techniques especially in fault diagnosis [167]. The integration of deep learning methods also brings a new perspective to predict the health of the machine. Convolutional neural networks work based on the idea of the selection of best local features with a feature extraction method in order to extract useful raw signals. Being a class of the neural networks, recurrent neural networks compose the connections between neurons of the input parameters to demonstrate the dynamic behavior of the network. Thus, it is possible to generate a map and compose a memory by keeping the momentary situation of the neural network using a back propagation algorithm [165,168]. The structure of the artificial neural network model is demonstrated in Figure 12.

In the particular example of this paper, FW prediction via artificial neural network in turning operations using different types of materials has been an attractive issue in the past. In references [35,82,83,84,85,86], artificial-neural-network-based TW prediction was successfully carried out and highly recommended. D’Addona et al. [83] performed a cognitive modeling of TW for Inconel 718 to obtain better tool life. They used neural network model for estimation of TW progression and results obtained with low prediction errors. Pal et al. [86] proposed a TW monitoring system for optimum cutting conditions in turning of EN19 steel using a neural network approach. As a flexible and simple approach, neural network provided a robust prediction option in practice. Ojha and Dixit [85] presented a neural network approach for continuous monitoring of tool life in the shop floor during turning of steel. Since a large number of data are available, tool life can be estimated using this example. In articles [34,81,83] neural-network-based TW prediction was also carried out for H-13 steel, AISI 1045 and SAE 6150 steel successfully.

### 5.2. Fuzzy Logic

**Figure 13:**
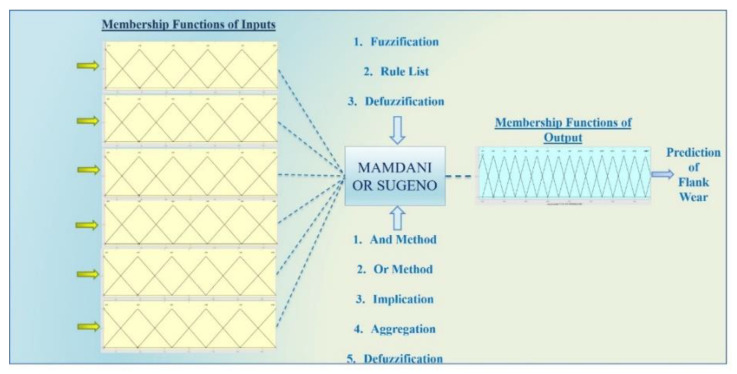
The structure of fuzzy logic model [5].

Fuzzy logic uses membership functions to link the input and output parameters via predetermined rule list. It is actually a reasoning-based methodology provides to estimate the desired parameter from 0 to 1 possibility. Human observation and the way of thinking is the example for development of the fuzzy logic approach. Fuzzification, rule editor and defuzzification processes are respectively applied to input data values. At first, the range of membership functions are defined with the training of input values; then, using rules, inputs and outputs are linked to each other, and lastly, fuzzy terms are converted to numerical values again with defuzzification. There are two basic fuzzy types, as Mamdani and Sugeno, in the modeling interface of fuzzy logic. In the prediction phase, methods should be identified, namely, and, or, implication, aggregation and defuzzification. The membership functions of inputs and outputs, type of fuzzy and decision methods are demonstrated in Figure 13. In [5], in addition to cutting parameters, AE and tool tip temperature were implemented to fuzzy inference model for predicting FW. According to the results, fuzzy inference system was successful in estimating the FW within a high success rate. Kuo [84] proposed a fuzzy logic based prediction of FW using CFs, vibration and AE data. It was reported that fuzzy approach was very promising.

### 5.3. Hidden Markov Model

**Figure 14:**
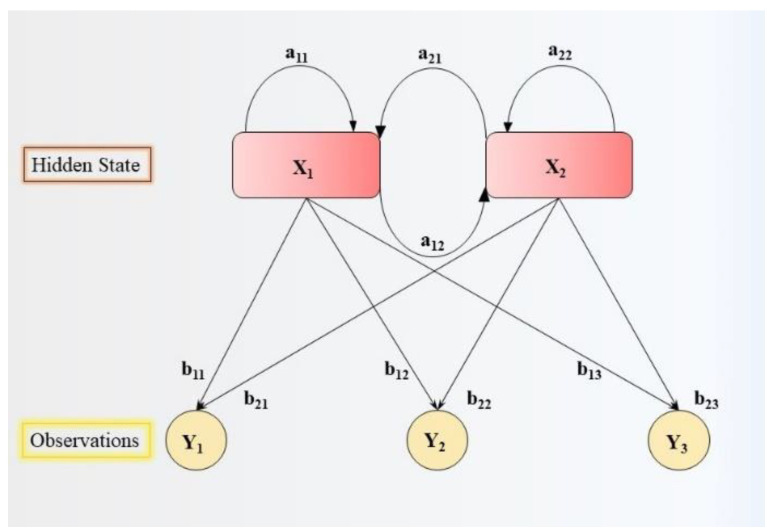
The structure of hidden Markov model.

Hidden Markov model is based on an observation strategy for the modeled system, which proposes to learn it via Markov process. Being another process, behavior is assumed as dependent and should be handled separately. Markov process is evaluated as a hidden or unobservable condition using the actual data in the system, which further ensures the validity of the result [162]. The usage of dependent factors for the recognition of observable events becomes useful for the ones improper to directly observe. Hidden Markov model enables to implement several software programs for modeling, which can be evaluated in a variety of processes, especially in machining. The structure of hidden Markov model is shown in Figure 14. Scheffer et al. [60] implemented neural networks and hidden Markov models as a comparative study in monitoring of TW. In turning of aluminum alloy, a comprehensive comparison was performed, and neural network was found as capable of performing continuous estimations; however, if the problem to solve is defined well, hidden Markov properly estimates TW. There were a handful of studies about hidden Markov model in machining operations, and a few of them are about TW prediction during turning. 

### 5.4. Support Vector Machine

**Figure 15:**
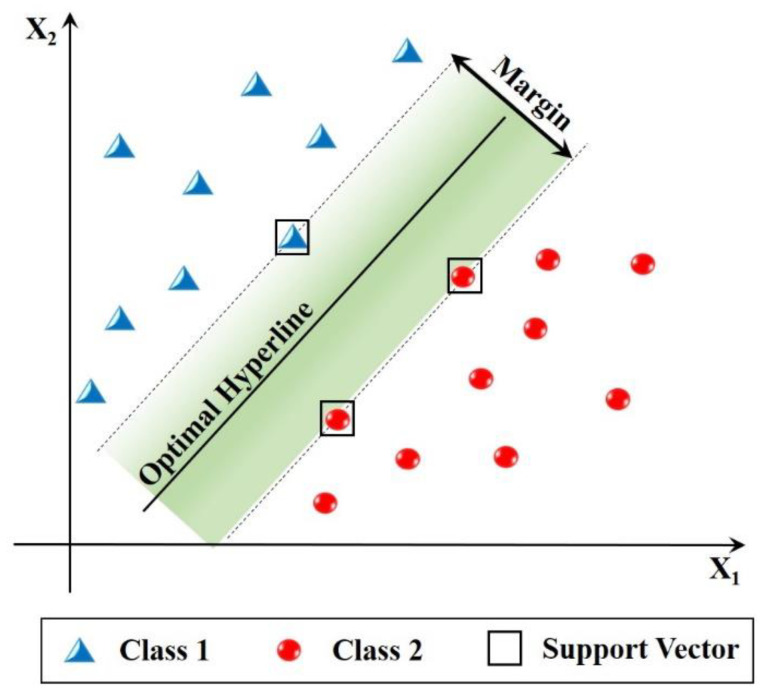
The structure of support vector machine.

Support vector machine is a supervised classification method in machine learning applications starting with the separable data at first, then modifying it to solve the problems having non-separable data [88]. As it is demonstrated schematically in Figure 15, data points are placed on a plane separated by a hyperplane, which are called support vectors. The position and direction of the hyperplane is determined by the support vectors which are closer to hyperplane. Some data points can be deleted from the plane, which aims to maximize margin and shape the hyperplane. The main purpose is to build the support vector machine by giving a form to this plane [169]. Despite traditional training methods, the support vector machine uses structural risk minimization instead of empirical risk minimization [87]. A principal advantage of this method is the capability to handle large data, since the magnitude of data have no important effect on the performance of the machine [170]. Widodo and Bo-Suk [87] proposed a support vector machine based TCMS in addition to fault diagnosis in a review. Sun et al. [88] presented a support vector machine approach to multi-classification of TW as sharp, usable and worn types via AE sensor. They used ASSAB705 and ASSAB760 steels for turning operation. Results indicated that this method can effectively reduce manufacturing loss with estimating tool life. A study from Brezak et al. [89], feed CF, AE and feed current signals were utilized to estimate TW. By this multi-sensor approach, the error was reduced by classifying the TW to clearly identify the tool condition. Kong et al. [90] used also support vector machine in prediction of TW in turning of steel. A dynamometer was utilized to model the machine and comparison was performed with other methods and support vector machine. Li et al. [91] performed force based TCM in turning for modeling FW. Experimental results showed that the prediction accuracy can be very high in predicting FW and possible tool failure.

### 5.5. Adaptive Network Based Fuzzy Inference System

**Figure 16:**
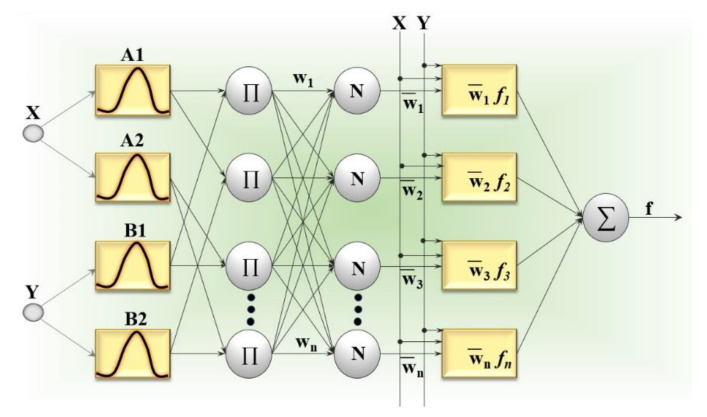
The structure of adaptive network based fuzzy inference system.

Adaptive network based fuzzy inference system is a combined neural network and fuzzy system which is based on extraction of fuzzy rules for every layer of neural network model. The structure of the adaptive network based fuzzy inference system is indicated in Figure 16. Gajate et al. [92], Lo [93], Xu et al. [94], Sharma et al. [95], Azmi [96], Liu et al. [97] and Rizal et al. [98] applied adaptive network based fuzzy inference system to turning operation for the purpose of TW monitoring and prediction with minimum error. A general consensus was observed in the studies in open literature: Adaptive network based fuzzy inference system could be effectively implemented in TCMS.

## 6. Discussion

Turning is a widespread machining operation that can efficiently remove chips from a rotating and cylindrical workpiece. Cutting tools are the main components of an entire production chain dealing with the workpiece which is considered as the ultimate aim of manufacturing. Turning tools are employed to change with new ones before reaching wear limit for healthier and low-cost operation. Tool breakage is a devastating event causing diverse damages such as machine downtime, workpiece deterioration and increased total costs. Unexpected tool breakage and usage of a cutting tool in lifetime requires TCMS that can diagnose the tool condition with sensor systems or pattern recognition approaches. Indirect TCMS are more suitable for industrial applications because of their applicability and sustainability and for being economical. Besides, the papers which have motivation to correlate the sensor signals with tool FW using indirect TCMS are included in this review. A general outline was performed on indirect TCMS for turning operations in this study for the last two decades. Advanced monitoring systems and their integration to manufacturing systems serve as a supportive component for production of complex parts. These systems enable detection of errors in cutting tools and workpieces for higher accuracy. Moreover, it is possible to set communications between sensors and machine tools to make decision for the health of the machining. Software based solutions can be obtained in this manner, which is a better approach for the management and supply chain in a production platform. In the future, it would be also possible to integrate different combination of advanced cutting tools and new generation materials. The experience on machining to date is a guide for this purpose, but there is a requirement for advanced artificial intelligence systems and their successful integration to manufacturing processes. The selection of appropriate signal processing and evaluation methods after choice and accomplished integration of sensor systems is noteworthy. There is need for complete and multi-disciplinary information on robust and high accuracy monitoring systems. TCMS based systems became reliable auxiliary especially in turning operations enhancing the quality of the workpiece, tool life and productivity. Besides, further applications on monitoring of cutting tool can be improved to adaptive control systems for online decision making. The following implications can be derived according to authors:

- Necessity: Basically, online monitoring of tool condition seems highly necessary to prevent unexpected developments, better component quality and optimized cutting conditions. A certain investment on TCMS provides more efficient and low-cost manufacturing in industry.
- Purpose: Generally, any improvement in the field of optimization can be one of the purposes of TCMS such as surface quality, dimensional accuracy, tool life, consumed power and energy, manufacturing time, manufacturing costs, idle time and waste material. Moreover, optimum cutting conditions can be obtained via TCMS, which will fulfill the aforementioned purposes within a particular quality.
- Primary advantages: Online monitoring of tool condition provides avenues to interfere the operation instantly with qualified equipment. Artificial-intelligence-based monitoring acts as a decision making mechanism rather than operator and makes deductions with high reliability.
- Additional advantages: Beyond monitoring machining conditions, these systems provide significant data source and optimized parameters for further usage. Besides, healthier turning conditions for operators can be obtained by preventing accidents. If TCMS is supported with supplementary device such as withdrawal mechanism; the intervention can be performed without operator control.
- Coverage and context: Especially in machining operations, for turning, drilling, milling, etc., TCMS proved that more accurate and sensitive manufacturing can be obtained. However, proper sensor systems can be integrated to any manufacturing technology and successfully applied.
- Drawback and deficiency: There is a need for investment cost to meet sensor systems, data acquisition equipment and software for data processing and recording. Multiple sensors can detect the system errors more accurately and predict tool condition with more sensitivity. It is an important issue to determine the number of sensors because of bringing additional financial worries.
- Recommendation: A universal approach should be developed instead of a certain pair of tool and workpiece investigation for each study. That is why the relationship between process parameters and TW should be analyzed and stated in detail. Description of sensor fusion must be clarified and generalized for robust, inexpensive and intelligent monitoring systems.
- Previous research work: In the far past of turning operations, a series of cutting tool materials have been applied to newly developed workpiece materials to achieve better machinability. With each of work material produced, it was intended to solve the several industrial and social problems, however innovations introduced new issues. In order to overcome these problems of each period, technological approaches presented from manufacturers and researchers such as new tool geometry, developed machine tools, different cooling technologies, the latest tool materials etc. As mentioned before, each of these initiatives accompanied mysteries and questions. TCMS evolved in time for different types of problems, unexpected failures, industrial accidents, to control the new technology and came to modern day. Even though it seems with the name “tool condition monitoring”, the system monitors the machine tool and workpiece. The basic structure of this system is available to integrate modern hardware and software components. Therefore, their existence provided a reliable manufacturing in the past and of course, will be the most important assistance in the future.

## 7. Critical Analysis and Trends

Mentioned topics for sensors and signal processing methods are handled and evaluated one by one in this paper. It should be noted that there is necessity to compose these hardware and software sources to follow future trends. Not only for a sensor and an artificial intelligence method but also using more than one sensor to obtain sensor fusion integrating hybrid decision making methods for a robust process. For that purpose, Industry 4.0 leads in industry with increasing expectations because of its extensive content which covers the intelligent manufacturing, smart manufacturing, sustainable manufacturing and digital manufacturing. Gathering these methods is just the beginning of the period. With newly discovered materials and developed models, previously proposed methods need updating especially for avoiding excessive costs, idle time, material losses and hard to recover mistakes. To date, all proposed methods addressed the technology in some way however with increasing interaction between humans and machines should direct it to develop faster and efficient. In the perspective of machining technologies, TCMS exist in a significant position providing reliable information about complex machining operations.

The prominent sensors have been considered as dynamometer, AE, temperature and accelerometer with their success in detection and preferability in the field of TCMS in turning. Almost all of these sensors are expensive and increase initial investment costs. However, advances and complications in machining technologies make these sensors essential, applicable, tenable and sustainable for online monitoring. Clearly, their quick response time and easy to implement structure is attractive to solve the sophistication while turning. For improved diagnosis and prognostics, the accurate implementation of decision-making methods for different types of sensors is notable. This situation generates a problem that there is need researchers represent different disciplines for more profound analysis during preprocess and post process. An important issue is fast convergence of the parameters for optimal values which can be possible using emerging data analysis methods. The integration of different types of data for an ultimate decision about the operation should be performed. Another challenging issue is collecting data together and analysis them which require comprehensive information that entail the utilization of data communication for health monitoring. In order to practice the prognostic health management with intelligent systems, the emerging technologies in the field namely edge computing, machine learning integrated embedded devices should be used. These systems monitor the operation with advanced software components for sustainability of the production while managing cost and safety. High technology supported manufacturing systems ensures the diagnosis of major errors affecting the dimensions of the workpiece and reducing remaining useful lifetime of the cutting tool. Generally, condition-based maintenance systems preserve the available situation which is predetermined by operator accepted that as the optimal condition. Eventually, for the purpose of the Industry 4.0, a holistic approach is required to avoid a variety of defects and breakdowns during the turning operation.

Artificial neural networks and ANFIS applications have been utilized satisfactorily in turning operations for FW as it can be clearly seen. These methods were first among the others which demonstrated the success of neural-networks-based prediction and monitoring. With this survey, it is intended to show the primary applications on sensors and decision-making technologies, their advantages and drawbacks. It is suggested that a specific TCMS equipped with correct sensors, signal processing method and classification is the most developed one for a specific work material. That drives the researchers to investigate each sensor feature and signal processing method in detail.

## 8. Conclusions

Despite there being important initiatives to evaluate TW, CFs, vibration, AE and SR, analysis, modeling and optimization were performed individually in some studies. On the other hand, a number of papers published in the field endeavored to estimate process parameters accurately. To date, important work has been carried out to correlate TW and sensorial data in turning. Thus, a critical review should be performed for TCMS to explain the relationship between sensorial data and quality characteristics, for example, TW. The following conclusions were drawn from this exhaustive literature review:

- The cross interaction between cutting parameters, in addition to the effect of TW mechanisms and TW types, makes cutting operation complex. Considering turning, single cutter is exposed to high mechanical, chemical and thermal loads which lead to different wear developments. FW is accepted as the main tool life criteria since it shows progress on the flank face and weakens the main cutting edge. Therefore, it is required to analyze in detail the FW especially with sensor systems to investigate their correlation. In this way, online tracking and detection of the condition of wear can be determined and further prevention of failure can be possible.
- The estimation of FW is a difficult task due to the time variant and non-linear structure of machining processes. This challenge pushes the researchers to observe the momentary alterations and protect the cutting tool from harsh conditions. Having a long history, TCMS served as an information source with developing systems. A subsection of TCMS is indirect systems, which are easy to implement and which provide effective solutions, when the previous papers are considered.
- As each innovation brings some inconveniences, the drawbacks are due to lack of knowledge, supplementary payment and possibility of inefficiency. Indirect TCMS presents valuable contributions such as capability of using cutting tools in their remaining useful life. This process can be managed by optimizing the other operation variables. Eventually, this technology offers a facility that brings multiple optimization of cutting variables along with the ultimate aim of the process parameters.
- The summarized methods belonging to signal processing and sensor systems prove the significance of different applications in order to solve various machinability problems related with TW. Considering the literature, a problem may be solved with a variety of techniques and with one way in some situations. That drives the researchers to find the correlations between variables and FW.
- As outlined, each sensor has some advantages and disadvantages; however, most of them develop the monitoring system enduring the tough conditions of machining for a long time. Thus, proper selection, integration and usage of a sensor or a group of sensors make the high costs of this investment tolerable. Manufacturing technologies extend their facilities with labor, economy, and engineering information in order to reach the goal of Industry 4.0. This committed literature review shows the importance of indirect TCMS for reaching the objective of Industry 4.0.

This paper focused on summarizing the relationship between indirect TCMS sensors and FW. Published papers in the last two decades in this area were outlined, data acquisition, signal processing and decision making methods were also investigated. The research can be carried out for milling and drilling operations and other types of TW.

## Figures and Tables

**Table 1:** Publications that used different methods in indirect tool condition monitoring system.

Method	Cutting Forces	Acoustic Emission	Vibration	Temperature	Sound	Current	Image Processing
Publications	[4,35,36,37,38,39,40,41,42,43,44,45,46]	[4,5,43,47,48,49,50,51,52,53]	[5,33,36,40,50,54,55,56,57,58,59,60]	[31,39,48,61,62,63,64,65,66,67,68]	[69,70,71,72,73]	[5,6,73,74,75]	[72,76,77,78,79,80,81]

**Table 2:** Publications that used decision making methods in indirect tool condition monitoring system.

Method	Artificial Neural Network	Fuzzy Inference System	Hidden Markov Model	Support Vector Machine	Adaptive Network Based Fuzzy Inference System
Publications	[35,82,83,84,85,86]	[5,84]	[60]	[87,88,89,90,91]	[92,93,94,95,96,97,98]

## Data Availability

Data sharing not applicable.

## Nomenclature

TCMS	Tool Condition Monitoring System
TCM	Tool Condition Monitoring
SR	Surface Roughness
CF	Cutting Force
AE	Acoustic Emission
TW	Tool Wear
FW	Flank Wear
